# Intersectionality of sex and race in COVID-19 mortality and vaccination inequities in Massachusetts

**DOI:** 10.1186/s12889-024-20340-z

**Published:** 2024-10-29

**Authors:** Allison Boretsky, Victoria Fisher, Nadia N. Abuelezam

**Affiliations:** Boston College Connell School of Nursing, Maloney Hall, 140 Commonwealth Ave, Chestnut Hill, MA 02467 USA

**Keywords:** COVID-19, Intersectionality, Vaccination, Health disparities

## Abstract

**Background:**

Inequities in COVID-19 incidence, morbidity, and mortality between racial and ethnic groups in the United States (U.S.) have been documented since the start of the pandemic in early 2020. Similarly, disparities by sex for COVID-19 morbidity and mortality have emerged, with men dying at a higher rate than women. Little research has been done to understand how the intersection of sex and race impacts COVID-19 inequities in Massachusetts (MA). This cross-sectional study examined how COVID-19 mortality rates (February 2020- May 2023) and vaccination rates (December 2020-February 2023) varied by sex across racial groups in MA.

**Methods:**

Using Massachusetts Department of Public Health data of all COVID-19 mortality cases and primary series vaccinations in MA from 2020 to 2023, we calculated both age-specific and age-adjusted COVID-19 mortality rates in order to account for differences in age distributions across sex-race groups.

**Results:**

Overall, men across all age-race groups consistently had a higher mortality rate compared to their female counterparts. The age-standardized mortality rate difference between White men and White women is the smallest, with the rate for White men being 1.3 times higher than White women. The age-standardized mortality rate between Hispanic men and Hispanic women varies the largest, with the rate for Hispanic men being 1.7 times higher than Hispanic women. Notably, Black women and White women have similar vaccination rates, yet the age-standardized mortality rate for Black women is 1.4 times the rate of White women.

**Conclusions:**

Our findings show that there are disparities at the intersection of sex and race for COVID-19 mortality and vaccination in MA. This highlights the importance for targeted COVID-19 interventions at the intersection of sex and race and the need for detailed COVID-19 reporting by sex within race groups.

## Background

As variants of the COVID-19 virus continue to impact life, COVID-19 inequities, in rates of infection, complications, and mortality, are becoming more apparent across racial and ethnic lines [1]. Among the 103.4 million confirmed COVID-19 cases in the United States (U.S.) (as of November, 2023), 1.14 million lives have been lost since the start of the pandemic due to the virus [2]. The Black community has experienced both higher rates of COVID-19 mortality and hospitalizations compared to other racial groups in the U.S [3]. Similarly, disparities by sex for COVID-19 morbidity and mortality have emerged, with most men dying at a higher rate than women across racial groups [4]. The COVID-19 pandemic has brought attention to existing gaps in the U.S. health system for the most vulnerable populations, especially women of color [3, 5]. As a result of historical structural racism and sexism, women of color face the compounded harms and challenges associated with both their gender and race and/or ethnicity. Women, particularly women of color, are more likely than men to live in poverty, increasing their risk of food insecurity during a time when both grocery stores and food banks are experiencing shortages [6]. Structural racism and sexism both contribute to health inequalities in the era of COVID-19. These systems of oppression lead to restricted access of essential healthcare services, affordable and quality produce, housing, and environmental exposures, contributing to adverse health outcomes amongst individuals [7]. However, few studies examine the intersectionality of sex across racial-ethnic identities in relation to COVID-19 outcomes.

Public health research often examines each system of oppression independently, impeding interventions to improve the health of people who exist within “diverse realms of experiences [8].” This leads to fewer interventions for vulnerable populations and forces those at the intersection of these identities (i.e. Black women) and researchers to choose a group (i.e. women or Black) with which they associate [9]. This unique situation isolates people of intersecting identities, obscuring their lived experiences. Unfortunately, public health research that considers an intersectional lens is rare [10]. Intersectionality is a theoretical framework used for understanding how multiple social identities that shape an individual’s lived experience, such as age, race, gender, and socioeconomic status, intersect with various systems of privilege and oppression (i.e., racism, sexism, classism, ageism) that exist in society [9, 10]. People can occupy multiple identities that intersect with structural forces, leading to inequitable health outcomes [9].

Oftentimes, studies distinguish between “women and minorities,” completely ignoring the fact that individuals exist at the intersection of these two identities [10]. This prevents the discovery of crucial information and public health interventions for individuals at these intersections, such as racially and ethnically minoritized women. A lack of research that specifically addresses systems of oppression such as racism, sexism, and ageism and their intersection is further evidence that intersectionality theory needs to be prioritized in public health [10].

The development of the COVID-19 vaccine provided an ostensibly universal intervention in the fight against the virus. However, vaccination rates have varied by racial and ethnic groups in the U.S [7]. In the 2022 State Health System Performance Analysis, Massachusetts (MA) ranked second out of all U.S. states on the basis of overall performance across 56 measures of health care access and quality, including health outcomes during the COVID-19 pandemic in 2020 [11]. Nearly 85% of the total MA population has completed their primary COVID-19 vaccination series, making it one of the highest vaccinated states in the U.S [12]. Despite efforts to encourage vaccine uptake, gaps by race still exist within MA [13]. In both MA and the U.S. broadly, those who identified as Black and Hispanic/Latino were less likely to receive the COVID-19 vaccine compared to their White, non-Hispanic counterparts [12–14]. To our knowledge, no prior work examines vaccine uptake by sex across racial-ethnic identities in MA. Analyzing vaccine data for MA helps identify inequities in vaccine distribution, uptake, and hesitancy by race and ethnicity and sex, allowing for targeted solutions and an understanding of how inequities function in a high performing health-system state.

There is limited research in few U.S. states that explores the intersection of sex across racial-ethnic identities in regards to COVID-19 mortality and little information relating to MA. Using data from the Massachusetts Department of Public Health, this cross-sectional study analyzes how COVID-19 mortality rates (February 2020- May 2023) and vaccination rates (December 2020-February 2023) varied by sex across racial-ethnic identities in MA.

## Methods

### Data sources

MA COVID-19 mortality and case data was collected by the Massachusetts Department of Public Health (MDPH) and Massachusetts Virtual Epidemiologic Network [15]. MA COVID-19 vaccination data was collected by MDPH and the Massachusetts Immunization Information System (MIIS) [16]. The Massachusetts government does not report COVID-19 mortality and vaccination data aggregated by race and ethnicity, sex, age, and gender, so aggregated data for this analysis was specially requested and obtained. Mortality data included all confirmed and probable cases from February 2020 to May 2023 and vaccination data included all recorded primary series COVID-19 vaccinations from December 2020 to February 2023. Study periods for mortality and vaccination differ due to differences in available data. COVID-19 vaccines did not become available to the public until December 2020 [17]. For mortality, all MA confirmed and probable mortality cases obtained by the MDPH (from February 2020- May 2023) were used. The American Community Survey (ACS) 5-Year Estimates Public Use Microdata Sample Vintage 2021 population estimates were used for all denominators [18]. The ACS 5-year population estimates are averages of population characteristics for a five-year period [19]. The ACS 5-year estimates are the most reliable and precise of the ACS period estimates [19]. The ACS 2021 5 year estimates were used as the benchmark population to which we standardized our rates due to inaccuracies reported by the Census Bureau for the 2020 1-year ACS estimates as a result of the pandemic. Potential limitations to using the 2021 year estimate includes not capturing the estimated change of populations from year to year; however, the ACS 5 year estimates describe the average characteristics for that 5-year time period [19].

### Exposures

Many covariates, including race, age, and sex, influence lived experiences and, thus, vulnerability to COVID-19 exposure and outcomes. The Census Bureau defines race as an individual’s self-identification with one or more social groups, which can be selected as American Indian or Alaska Native, Asian, Black or African American, Native Hawaiian or Other Pacific Islander, or some other race [14]. Ethnicity data, collected independently from race, is determined by whether individuals consider themselves to be of Hispanic origin. The MIIS did not store gender data; therefore, in regards to gender identity, all non-binary data and responses where sex was not reported are included in the Unknown/Other category.

For COVID-19 mortality, racial and ethnic categories with non-missing mortality data included in this study were: Hispanic; non-Hispanic (NH) Asian; NH Black; NH Other race; Unknown; and NH White. Sex was reported as female, male, transgender, and unknown [15]. “Other race, non-Hispanic” included individuals who identified as a race that are not the categories explicitly provided as well as individuals who identify as multi-race [20]. In data reported prior to December 30, 2020, these two categories were captured under Other race, non-Hispanic [20].

For the vaccination analysis, original data source’s race and ethnicity categories included Black, White, Asian, American Indian/Alaska Native (AI/AN), Unknown/Other, Multi, as well as ethnicity separated by Hispanic and non-Hispanic [16]. “Other” included responses that did not fit into the listed categories, as well as non-responses or “prefer not to say.” The MDPH defined “multi race” as those who selected more than one race, other than unknown or other. “Unknown” included records from providers whose software did not report or collect race and ethnicity data or when no race or ethnicity was selected or recorded. Total counts for racial groups reported were for individuals who identified as non-Hispanic/Latino, whereas numbers for Hispanic/Latino were reported for all who identified as Hispanic/Latino regardless of race.

### Outcomes

Confirmed COVID-19 mortality was defined by the CDC as a person who died “within 30 days of their first positive molecular test and their manner of death is marked as natural; or COVID-19 is listed on their death certificate following a positive molecular test regardless of time since diagnosis.” [12] Probable mortality was defined by the CDC as “a death that meets the clinical criteria and epidemiologic linkage with no confirmatory laboratory testing performed for COVID-19.” [21] Guidelines for including probable and confirmed COVID-19 mortality were established by the CDC. In mortality cases where it is suspected or likely (e.g., the circumstances are compelling within a reasonable degree of certainty) but a definite diagnosis of COVID-19 cannot be made, it is acceptable to report COVID-19 on a death certificate as “probable” or “presumed.” [22].

Our vaccination analysis only considered those who had completed their primary COVID-19 vaccination series. The primary vaccination series was defined as individuals who received two doses in a 2-dose series (original Pfizer, original Moderna, or Novavax), or a complete single-dose vaccine (bivalent Pfizer, bivalent Moderna, or Johnson & Johnson) [23].

### Statistical analysis

Age-specific and age-adjusted COVID-19 mortality rates were calculated in order to account for differences in age distributions across sex-race groups. Age-specific mortality rates were calculated by dividing the number of deaths in each age-sex-race group by the total population for each sex-race group in MA and reported per 100,000 individuals [24]. Age-adjusted rates were calculated using cited age-standardized mortality rate (ASMR) methods with the 2000 U.S. Standard Populations [25]. The American Community Survey 5-Year Estimates Public Use Microdata Sample Vintage 2021 population estimates were used for all denominators [18, 26]. 95% confidence intervals for age-adjusted rates were estimated using the standard error of age-standardized mortality rates [27]. Rate ratios for age-standardized mortality rates were calculated between sexes within race groups and across races within sex groups. Rate ratios were calculated using the standard method of calculating the ratio between two rates using the reference group as the denominator [28]. 95% confidence intervals for rate ratios were estimated using sampling variance [29].

We use the NH Other race MA American Community Survey-5 year population estimate for 2021. Vaccination rates were reported as the total number of each sex-race group of interest who received at least one dose of the COVID-19 vaccine series out of the total population for each demographic group per 100,000. The American Community Survey 5-Year Estimates Public Use Microdata Sample Vintage 2021 population estimates were used for all denominators [18].

For both mortality and vaccination calculations in our analysis, Unknown/Other and transgender sex categories were excluded. For the purpose of our analysis, any unknowns, N/A, NH American Indian/Alaskan Native (AI/AN), NH Native Hawaiian/ Pacific Islander (NH/PI) and missing variables were excluded from mortality calculations. Additionally, any unknown/Other race was excluded in the vaccination analysis. Variables where sufficient data was not provided to perform necessary calculations due to privacy concerns, generally referencing cell counts of fewer than five persons, were excluded, a finding particularly true for the AI/AN and NH/PI categories. Data analysis was completed using RStudio (2023.06.2 + 561) and Excel.

## Results

In total, 24,649 COVID-19 mortality cases and 5,632,526 primary series vaccinations were considered for our analysis between 2020 and 2023. We excluded 249,788 primary series vaccination cases due to unknown race/ethnicity and/or gender or insufficient counts. Our mortality study deaths were 0.355% and our vaccination study population was 80.6% of the total population of MA in 2021 (Table 1). In the mortality study deaths, 51.3% were men and 48.7% were women. Our mortality study deaths were majority White, non-Hispanic (73.1%, Table 1). In the total vaccination study population, 50.9% were women and 44.7% were men (Table 1). The missing percentage is composed of unknown and/or transgender individuals, which were excluded from our study. Our vaccination study population was majority White, non-Hispanic (67.4%, Table 1).

**Table 1:** Descriptive statistics of MA Mortality and Vaccination Study Population Feb 2020-May 2023

Variable			Total Count		% of Study Population		% of MA Population		
Total MA Population in 2021^a^				6,991,854		---		100%	
Mortality Study Deaths				24,649		100%		0.353	
*Sex*									
Male				12,640		51.3		0.181	
Female				12,009		48.7		0.172	
*Race*									
White, NH				18,026		73.1		0.258	
Asian, NH				650		2.64		0.009	
Black, NH				1457		5.91		0.021	
Hispanic				2226		9.03		0.032	
Other, NH				2290		9.29		0.033	
*Age Group*									
0–29				120		0.487		0.002	
30–49				695		2.82		0.010	
50–69				4578		18.6		0.065	
70+				19,256		78.1		0.275	
Vaccination Study Population				5,632,526		100%		80.6	
*Sex*									
Male				2,517,414		44.7		36.0	
Female				2,865,324		50.9		41.0	
*Race*									
White, NH				3,797,176		67.4		54.3	
Asian, NH				402,676		7.15		5.76	
Black, NH				360,278		6.40		5.15	
Hispanic				599,652		10.6		8.58	
Multi, NH				217,765		3.87		3.11	
AI/AN, NH				5,191		0.092		0.074	
^a^Total MA population according to the American Community Survey (ACS) 5-year Public Use Microdata Sample Vintage 2021 population estimates for each sex within race group									

### Mortality rates

**Fig. 1:**
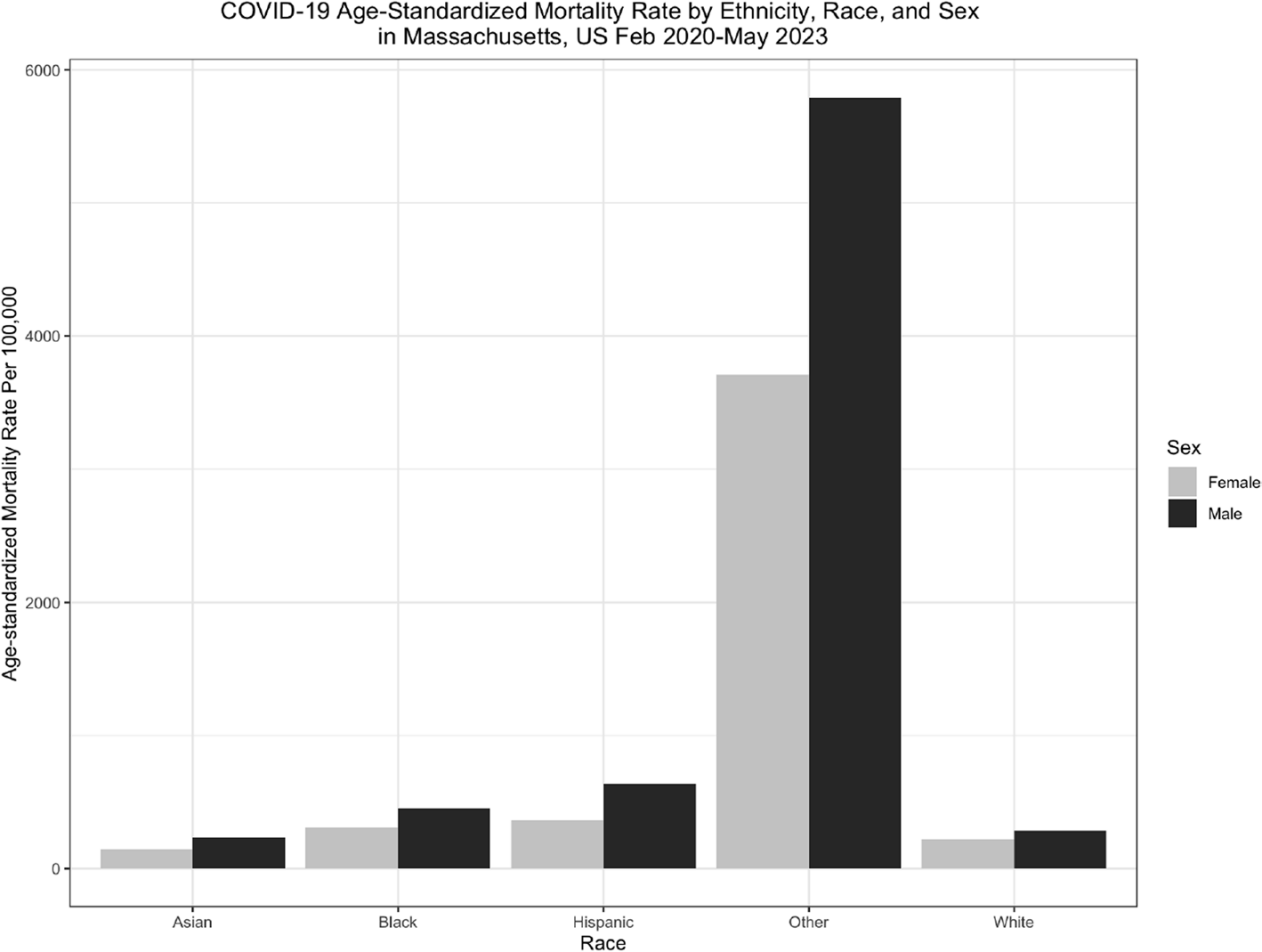
Age-standardized mortality rates are reported per 100,000 individuals, using the American Community Survey (ACS) 5-year Public Use Microdata Sample Vintage 2021 population estimates for each sex within race group. Within each race/ethnicity group, men had higher rates of age-standardized mortality compared to female counterparts

Overall, men across all age-race groups consistently had a higher COVID-19 mortality rate compared to their female counterparts (Fig. 1). To illustrate, in the 50–69 years age category, Asian men had a mortality rate of 223.75 per 100,000 (95% CI: 178.99, 268.51) while Asian women had a mortality rate of 99.07 per 100,000 (95% CI: 70.74, 127.39) (Table 2). The Other, NH race group (4579.61 per 100,000) had the highest age-standardized mortality rates when compared to all race/ethnicity groups. Within every sex-race stratum, the 70 + age group had the highest age-specific mortality rate (Table 2).

**Table 2:** COVID-19 Mortality Rate* by Age, Ethnicity, Race, and Sex in Massachusetts, US Feb 2020-May 2023

Race/Ethnicity			Sex											
			Female				Male				Total			
			Count	MA Population	Rate^a^ (95% CI)		Count	MA Population	Rate^a^ (95% CI)		Count	MA Population	Rate^a^ (95% CI)	
Asian, NH														
0–29 years			**	99,598	---		**	96,416	---		**	196,014	---	
30–49 years			10	86,057	11.62 (4.42, 18.82)		17	76,988	22.08 (11.58, 32.58)		27	163,045	16.56 (10.31, 22.81)	
50–69 years			47	47,443	99.07 (70.74, 127.39)		96	42,905	223.75 (178.99, 268.51)		143	90,348	158.28 (132.33, 184.22)	
70 + years			212	16,110	1315.95 (1138.81, 1493.10)		268	13,297	2015.49 (1774.19, 2256.80)		480	29,407	1632.26 (1486.24, 1778.29)	
Age Standardized Rate			269		143.03 (125.94, 160.13)		381		233.64 (210.18, 257.11)		650		184.59 (170.40, 198.78)	
Crude Rate			269	249,208	107.94 (95.04, 120.84)		381	229,606	165.94 (149.27, 182.60)		650	478,814	135.75 (125.32, 146.19)	
Black, NH														
0–29 years			2	101,781	---		11	101,821	10.80 (4.42, 17.19)		13	203,602	6.39 (2.91, 9.86)	
30–49 years			35	66,286	52.80 (35.31, 70.29)		52	65,357	79.56 (69.62, 89.51)		87	131,643	66.09 (52.20, 79.98)	
50–69 years			193	54,997	350.93 (301.42, 400.44)		258	49,563	520.55 (457.03, 584.07)		451	104,560	431.33 (391.52, 471.14)	
70 + years			472	19,129	2467.46 (2244.85, 2690.06)		434	12,293	3530.46 (3198.31, 3862.62)		906	31,422	2883.33 (2695.58, 3071.08)	
Age Standardized Rate			702		308.93 (286.07, 331.78)		755		449.98 (417.88, 482.07)		1457		367.96 (349.06, 386.85)	
Crude Rate			702	242,193	289.85 (268.41, 311.29)		755	229,034	329.65 (306.13, 353.16)		1457	471,227	309.19 (293.32, 325.07)	
Hispanic														
0–29 years			11	218,369	5.04 (2.06, 8.01)		28	228,601	12.25 (7.71, 16.79)		39	446,970	8.73 (5.99, 11.46)	
30–49 years			51	124,598	40.93 (29.70, 52.17)		124	125,605	98.72 (81.35, 116.10)		175	250,203	69.94 (59.58, 80.31)	
50–69 years			269	70,806	379.91 (334.51, 425.31)		418	62,623	667.49 (603.50, 731.48)		687	133,429	514.88 (476.38, 553.38)	
70 + years			621	20,284	3061.53 (2820.73, 3302.32)		704	13,548	5196.34 (4812.48, 5580.19)		1324	33,832	3916.41 (3705.53, 4127.29)	
Age Standardized Rate			952		366.65 (343.36, 389.94)		1274		636.97 (602.00, 671.95)		2226		480.68 (460.71, 500.65)	
Crude Rate			952	434,057	219.33 (205.39, 233.26)		1274	430,377	296.12 (279.76, 312.27)		2226	864,434	257.51 (246.81, 268.21)	
Other, NH														
0–29 years			7	14,051	---		5	13,635	---		12	27,686	43.34 (18.82, 67.87)	
30–49 years			34	9,417	361.05 (239.68, 482.41)		47	8,843	531.49 (379.54, 683.45)		81	18,260	443.59 (346.99, 540.20)	
50–69 years			175	7,213	2426.17 (2066.71, 2785.64)		288	6,630	4343.89 (3842.20, 4845.59)		463	13,843	3344.65 (3039.99, 3649.31)	
70 + years			850	2,501	33986.41 (31701.59, 36271.22)		884	1,692	52245.86 (48801.71, 55690.01)		1734	4,193	41354.64 (39408.13, 43301.14)	
Age Standardized Rate			1066		3709.09 (3486.43, 3931.75)		1224		5791.11 (5466.68, 6115.55)		2290		4579.61 (4392.04, 4767.18)	
Crude Rate			1066	33,182	3212.59 (3019.73, 3405.44)		1224	30,800	3974.03 (3751.39, 4196.66)		2290	63,982	3579.13 (3432.54, 3725.73)	
White, NH														
0–29 years			23	777,784	2.96 (1.75, 4.17)		33	795,561	4.15 (2.97, 5.33)		56	1,573,345	3.56 (2.63, 4.49)	
30–49 years			115	597,517	19.25 (15.73, 22.76)		210	589,632	35.62 (32.05, 39.18)		325	1,187,149	27.38 (24.40, 30.35)	
50–69 years			1054	740,563	142.32 (133.73, 150.92)		1780	707,964	251.43 (239.74, 263.11)		2834	1,448,527	195.65 (188.44, 202.85)	
70 + years			7828	385,198	2032.20 (1987.18, 2077.22)		6983	282,590	2471.07 (2413.11, 2529.03)		14,811	667,788	2217.92 (2182.20, 2253.64)	
Age Standardized Rate			9020		220.55 (216.00, 225.10)		9006		286.58 (280.66, 292.50)		18,026		250.22 (246.57, 253.87)	
Crude Rate			9020	2,501,062	360.65 (353.20, 368.09)		9006	2,375,747	379.08 (371.25, 386.91)		18,026	4,876,809	369.63 (364.23, 375.02)	

Data Source: Massachusetts Virtual Epidemiologic Network

** Insufficient data

--- Rates for counts less than 10 have been excluded

*NH* Non-hispanic

^a^Rate per 100,000 individuals, using the American Community Survey (ACS) 5-year Public Use Microdata Sample Vintage 2021 population estimates for each sex within race group

Hispanic men had the second highest age-standardized mortality rate (636.97 per 100,000) compared to men in all race/ethnicity groups. Hispanic women had the second highest age-standardized mortality rate (366.65 per 100,000) compared to women in all race/ethnicity groups. Black men had a higher mortality rate compared to Black women (449.98 per 100,000 vs. 308.93 per 100,000). White men (286.58 per 100,000) and White women (220.55 per 100,000) had the second lowest mortality rate compared to their counterparts in other race/ethnicity groups. Notably, Asian men (233.64 per 100,000) and Asian women (143.03 per 100,000) had the lowest age-standardized mortality rates compared to their counterparts in all race/ethnicity groups (Table 2).

Asian females aged 70+ (99.07 per 100,000) have a significantly lower age-specific mortality compared to Asian males aged 70+ (223.75 per 100,000) (Table 2). Black females aged 30–49 (52.80 per 100,000) have an age-specific mortality rate that is higher than their White counterparts (19.25 per 100,000) (Table 2). Black males aged 0–29 (10.80 per 100,000) have an age-specific mortality that is more than double that of White males aged 0–29 (4.15 per 100,000) (Table 2).

### Vaccination rates

**Fig. 2:**
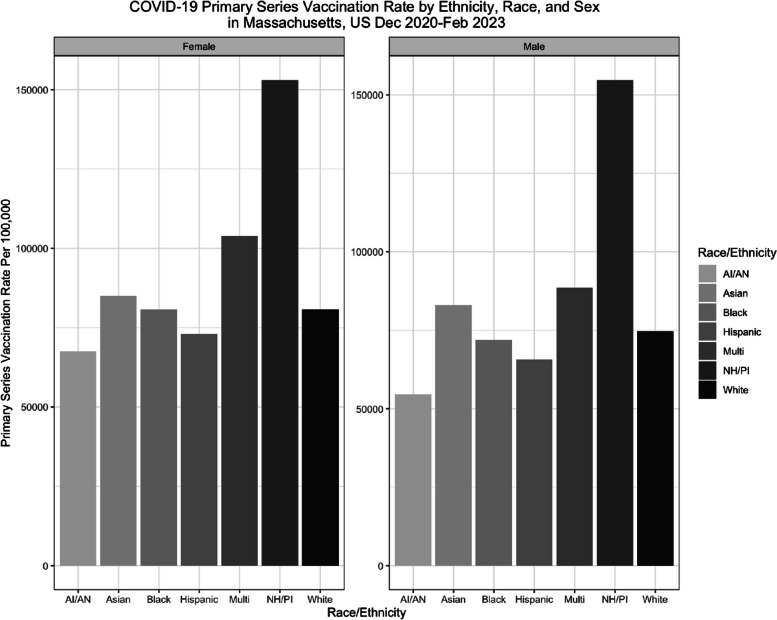
Vaccination Rates are reported as rate per 100,000 individuals, using the American Community Survey (ACS) 5-year Public Use Microdata Sample Vintage 2021 population estimates for each sex within race group NH/PI = Native-Hawaiian, Pacific Islander AI/AN = American Indians and Alaska Natives

Across all race-ethnicity groups, men had lower primary series vaccination rates compared to their female counterparts. Multi-race females (103,811.53 per 100,000) and multi-race males (88,548.94 per 100,000) had the highest vaccination rates out of all sex-race groups (Table 3). Black women had a higher average vaccination rate (80,759.97 per 100,000) compared to Black men (71,903.30 per 100,000) (Table 3). Asian men (83,000.44 per 100,000) and Asian women (85,110.43 per 100,000) had the second highest vaccination rates compared to their counterparts in each sex-race group (Table 3). Asian women had the highest average vaccination rate, excluding Multi-race men and women, (85,110.43 per 100,000) out of all sex-race groups (Table 3). Residents who identified as Hispanic had the lowest vaccination rate (69,369.32 per 100,000 total) compared to those who identified as White, Asian, or Black (Fig. 2).

**Table 3:** COVID-19 Primary Series Vaccination Rate^a^ by Race, Ethnicity, and Sex in Massachusetts, US December 2020 to February 2023

Race/Ethnicity		Sex								
		Female			Male			Total		
		Count	MA Population	Rate^a^	Count	MA Population	Rate^a^	Count	MA Population	Rate^a^
AI/AN, NH		2527	3740	67566.84	2664	4880	54590.16	5191	8620	60220.42
Asian, NH		212,102	249,208	85110.43	190,574	229,606	83000.44	402,676	478,814	84098.63
Black, NH		195,595	242,193	80759.97	164,683	229,034	71903.3	360,278	471,227	76455.30
Hispanic		316,674	434,057	72956.78	282,978	430,377	65751.19	599,652	864,434	69369.32
Multi, NH		118,205	113,865	103811.53	99,560	112,435	88548.94	217,765	226,300	96228.46
White, NH		2,020,221	2,501,062	80774.53	1,776,955	2,375,747	74795.63	3,797,176	4,876,809	77861.90

Data Source: Massachusetts Virtual Epidemiologic Network

*NH* Non-hispanic

^a^Rate per 100,000 individuals, using the American Community Survey (ACS) 5-year Public Use Microdata Sample Vintage 2021 population estimates for each sex within race group

## Discussion

Our findings show that there are disparities at the intersection of sex and race for COVID-19 mortality and vaccination rates in MA. Male and female rates across all race/ethnicity groups were quite different from each other. The age-standardized mortality rate difference between White men and White women is the smallest, with the rate for White men being 1.3 times higher than White women (Table 4). The age-standardized mortality rate between Hispanic men and Hispanic women varies the largest, with the rate for Hispanic men being 1.7 times higher than Hispanic women (Table 4). Notably, Black women and White women have similar vaccination rates, yet the age-standardized mortality rate for Black women is 1.4 times the rate of White women (Table 4). COVID-19 vaccination is shown to be effective at preventing serious COVID-19 complications and death [30]. The stark differences in mortality between Black women and White women is suggestive of the oppressive force of racism that underlies the health inequalities and barriers Black women face.

**Table 4:** Rate Ratios of COVID-19 Age-standardized Mortality Rates Using Various Reference Groups

	Ratio	CI (95%)
*Females as Reference Group*		
Asian, NH		
Male	1.6	1.3, 2.0
Black, NH		
Male	1.5	1.3, 1.7
Hispanic		
Male	1.7	1.5, 1.9
Other, NH		
Male	1.6	1.5, 1.7
White		
Male	1.3	1.1, 1.5
*White as Reference Group*		
Men		
Asian, NH	0.82	0.69, 0.97
Black, NH	1.6	1.4, 1.9
Hispanic	2.2	1.9, 2.5
Other, NH	20.2	17.9, 22.7
Women		
Asian, NH	0.65	0.52, 0.80
Black, NH	1.4	1.2, 1.7
Hispanic	1.7	1.4, 2.0
Other, NH	16.8	14.7, 19.2

Data Source: Massachusetts Virtual Epidemiologic Network

*NH* Non-hispanic

Within every race group, women had higher COVID-19 vaccinations rates compared to their male counterparts. One possible explanation for this is that a greater number of women had earlier access to the vaccine; in MA, female workers made up 77% of the front line health care workforce during the COVID-19 pandemic [31]. Massachusetts healthcare workers were first offered vaccines during Phase 1 of vaccine rollout in December of 2020, five months before it was available to the general public [32]. Social stigmas surrounding typical masculine roles may influence anti-vaccination tendencies and not prioritizing preventative health practices amongst men which may further explain the sex disparities in vaccination [33]. The intersection of structural racism and sexism that can create a general distrust towards medical staff may be influencing the sex-race disparities seen in COVID-19 vaccination [34]. At the intersection of these gender and race disparities seen in COVID-19 vaccination, men of color’s lack of COVID-19 vaccination is only augmented by their multifaceted identities [35]. Analysis of vaccine uptake by race and ethnicity and sex can highlight barriers to vaccination. Vaccine hesitancy and disparities in access are possible explanations for the lower rates of vaccination seen in U.S. minoritized racial and ethnic individuals during the initial phase of vaccine distribution [34]. Medical racism, lack of access to vaccines, and health providers can all impact vaccination rates within certain race and sex groups [5].

In MA, Black and Hispanic residents have inadequate healthcare coverage and access compared to their White counterparts [36]. Hispanic Massachusetts residents have long been plagued by social, economic, and educational inequalities that have only been heightened by the COVID-19 pandemic. Hispanic MA residents had a higher food insecurity rate compared to other Hispanic people in the U.S., as well as when compared to other racial and ethnic groups in MA [37]. People of color are at a greater risk of facing COVID-19 complications due to underlying social determinants of health and life-course outcomes that persist well beyond the scope of the COVID-19 pandemic. Age, male sex, obesity, and comorbidities are important determinants for severe disease, hospitalization, and mortality as well as social consequences and compounded stress of racism and sexism can lead to underlying health conditions. Intersectionality is crucial in understanding more about the most at-risk populations for COVID-19 exposure and the success of vaccination as an intervention [38]. Further research is needed to analyze the association between life course, pre-existing conditions, and COVID-19 outcomes.

Previous studies have yielded a similar conclusion to our analysis. A study conducted in Georgia and Michigan by Rushovich et al. concluded that Black men experience the highest COVID-19 mortality rates [39]. The study shows that men of all racial and ethnic groups are at higher risk of COVID-19 mortality compared to their female counterparts, and that both Black men and women have significantly higher COVID-19 mortality compared to their respective White counterparts [39]. Their findings also conclude that an intersectional framework for public health issues is crucial to revealing more complex patterns [39]. For example, in their analysis the age standardized COVID-19 mortality rate among identified Black females was 5.0 (95% CI: 4.4, 5.6) times the rate of White women in Michigan [39]. Comparatively, within the same study in Michigan, the COVID-19 mortality rate among White men was only 1.3 (95% CI: 1.2, 1.4) times the rate of White women [39]. This is much higher in magnitude than what our study revealed, which could be attributed to a shorter study period of only a few months in 2020 compared to our study which spans multiple years (2020–2023). Our longer study period is more likely to provide insight to patterns occurring throughout the entire course of the pandemic, rather than exposing inequalities first seen when COVID-19 emerged in the U.S [40–42]. Differences in state specific mortality and vaccination rates can be a result of differences in state policies, demographics, and environment. Previous studies show that Black, Hispanic, American Indian/Alaskan Native, and Native Hawaiian/Pacific Islander people have experienced higher rates of age-adjusted COVID-19 cases and deaths than White people [43]. There are many steps to take towards closing the gap between social inequities that exist in public health issues including recording detailed, disaggregated data, developing targeted interventions, and increasing access to resources [44].

Our study is not without limitations. Due to data suppression and privacy concerns, we were unable to adjust mortality rates for age in all race and gender groups. Second, ambiguous race definitions led to misclassification and variation in results of the “Other race” population [45]. The “Other race” category was not meant to generate large numbers; however, more than 1 in 7 people in the U.S. identify with this group [45]. The “Other” group is a catch-all for minoritized identities that prevents targeted interventions to specific groups in need. The large number of citizens who identify with the “Other race” and the underestimation of this category by the census results in a high magnitude of mortality seen in our study. Additionally, we found a discrepancy between the actual multi-race female population and the Census multi-race female population size. This is most likely due to varying definitions of the multi-race category, variation among individual self-reporting of race, and inconsistent reporting by providers, illustrating the need for more consistent definitions and data coding across public health institutions. Third, no definitive sex-race total population denominators exist, and rates vary depending on the population estimates used within calculations, leading to obscure results. While a range of public health organizations collect data, the data definitions and demographic categories are not consistent [46]. Within our own analysis, the racial categories varied between the vaccination and mortality data, where one dataset contained the “Multi” racial category and the other did not. The lack of standardized racial categories for all COVID-19 related data from MDPH makes it difficult to determine who is being represented and/or excluded across our indicators of interest. Our analysis exhibits how varying approaches to race and ethnicity data and definitions can lead to different conclusions about disparities.

There is an important need for using standardized race and ethnicity categories and definitions in health research [47]. Lack of transparent racial data and data disaggregation prevents interventions that strive for health equity [45]. Race is complex and the standard 5 + race categories do not resonate with many Americans nor capture detailed enough data [47]. For example, there is a need to break down the Asian race and Hispanic ethnicity categories, respectively. Based on our results, the question of whether or not high vaccination rates apply to one subgroup or are consistent throughout the entire Asian category is unclear. According to the 2010 U.S. Census, an Asian individual is defined as “a person who has origins in the Far East, Southeast Asia, or India [48].” Twenty-three million Asian Americans within the U.S identify with more than 20 different countries in East and Southeast Asia and the Indian subcontinent [48]. This broad category conceals potential underlying structures that could render one population more vulnerable to a particular outcome than another. This is relevant to our study because our results cannot conclude if all sub-groups classified within the “Asian” category truly have the lowest COVID-19 mortality out of all races and ethnicities. Classifications of race and ethnicity should be consistent across all datasets with unambiguous definitions [47]. Lack of uniform race and ethnicity definitions across public health departments can lead to varying numbers in race that impact data analysis and true identification of vulnerable populations.

The sex-race differences in vaccination seen in our results highlight the importance for targeted COVID-19 interventions and the need for detailed COVID-19 reporting by sex within race groups. This analysis demonstrates that a lack of standardized race and ethnicity categories creates difficulties in understanding how different groups are affected due to structural and systemic barriers [49]. The appropriate assessments of age, race, ethnicity, and gender and their intersections are crucial to public health research. Work that averages vaccination and/or mortality rates across both sex and race disguises important patterns that may help researchers understand the needs of minoritized groups at the intersection of multiple identities. Many government agencies and organizations design and allocate public health interventions based upon data containing racial and ethnic categories [47]. Without accurate results exposing how the intersection of identities and a multitude of social determinants, we cannot allocate resources efficiently and effectively. We acknowledge that we cannot treat entire sex-race groups as having an equal risk within a group; risk of COVID-19 mortality and vaccination outcomes vary by individual and can be influenced by things such as income, marriage status, occupation, employment status, etc. The differences in trends of sex-race-age groups we noted highlight how systems of oppression overlap in the U.S. to create health outcome differences that would otherwise be obscured by focusing on only one broader group.

## Data Availability

Code available in GitHub repository (https://github.com/allib28/Intersectionality-of-Sex-and-Race-in-COVID-19-Mortality-and-Vaccination-Inequities-in-Massachusetts.git) and de-identified data used for the current study or additional statistics derived from the data are available from the corresponding author on reasonable request with permission from Massachusetts Department of Public Health (MDPH).

## Abbreviations

U.S: United States
CDC: Center for Disease Control
MA: Massachusetts
MDPH: Massachusetts Department of Public Health
MIIS: Massachusetts Immunization Information System
PI: Native Hawaiian, Pacific Islander
AI/AN: American Indian/Alaska Native
ASMR: Age-standardized mortality rate
NH: Non-Hispanic
